# Supplementary material for: Improved analysis of (e)CLIP data with RCRUNCH yields a compendium of RNA-binding protein binding sites and motifs
Source: Genome Biol. 2023 Apr 17;24:77. doi: 10.1186/s13059-023-02913-0 (PMC10108518; doi:10.1186/s13059-023-02913-0)
Supplement: Supplementary file 1 — Additional file 1: Figure S1. Simulation of a CLIP experiment. Figure S2. Agreements of peaks identified between individual ENCODE samples. Figure S3. Similarity of de novo predicted motifs of different RBPs. Figure S4. RCRUNCH results for all ENCODE eCLIP data currently available. Figure S5. Evaluation of the model used to identify enriched regions. Figure S6. Binding events spanning splice junctions. Figure S7. Motif enrichments as a function of the number of top peaks. Figure S8. RCRUNCH application to PAR-CLIP data. [file 13059_2023_2913_MOESM1_ESM.docx]

# Supplementary Figures


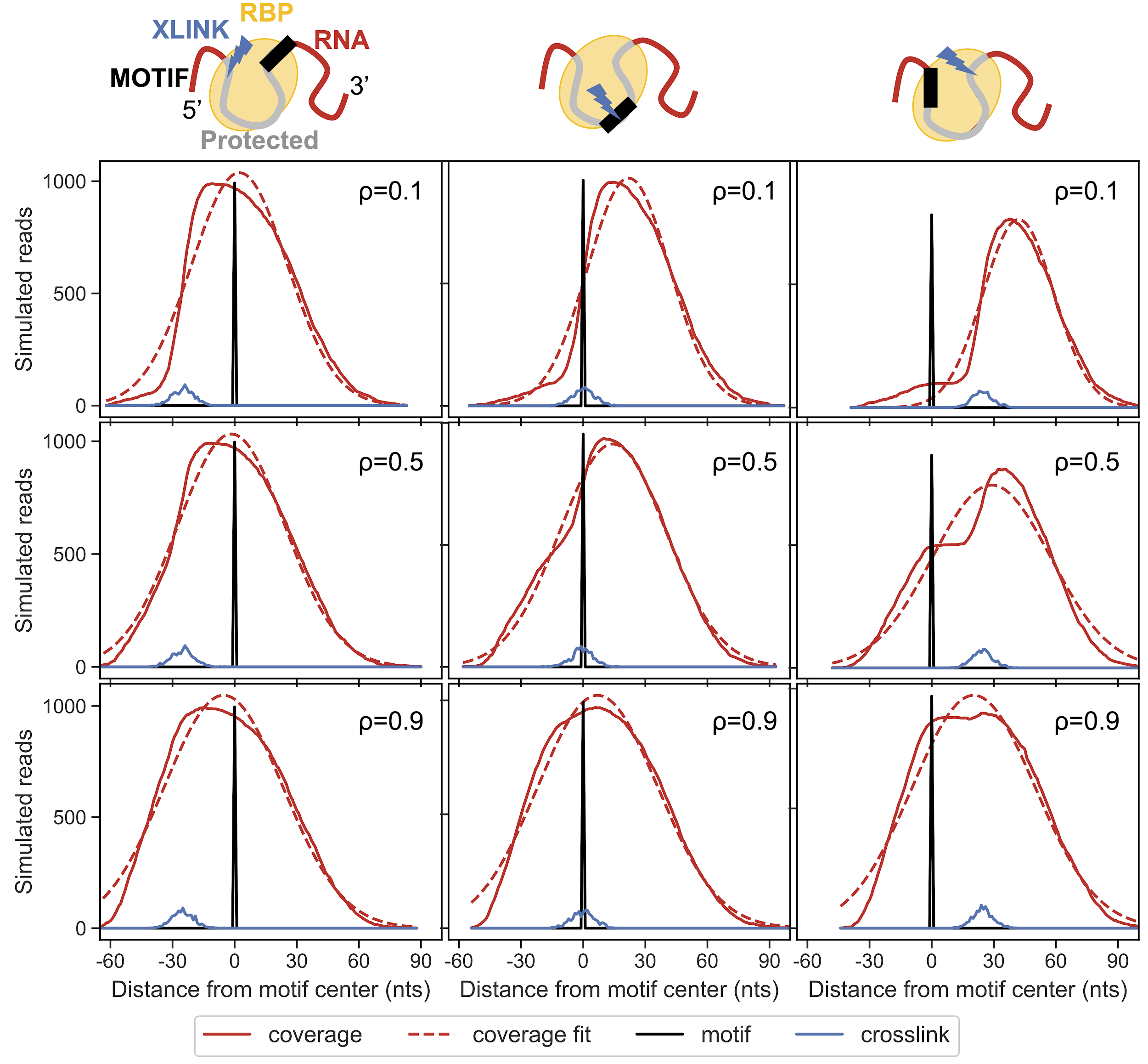


**Figure S1.** **Simulation of a CLIP experiment.** Upper schema: an RBP binding to its cognate motif, (black box), crosslinking to the RNA at the site indicated by the blue arrow, and protecting an extended region of the target (shown in gray) from digestion. From right to left, the relative position of the crosslink is changed relative to the motif position. The different columns correspond, as the schema shows, to different positions of the crosslink relative to the motif position. Each row corresponds to different probabilities of readthrough, varying from lower to higher from top to bottom.


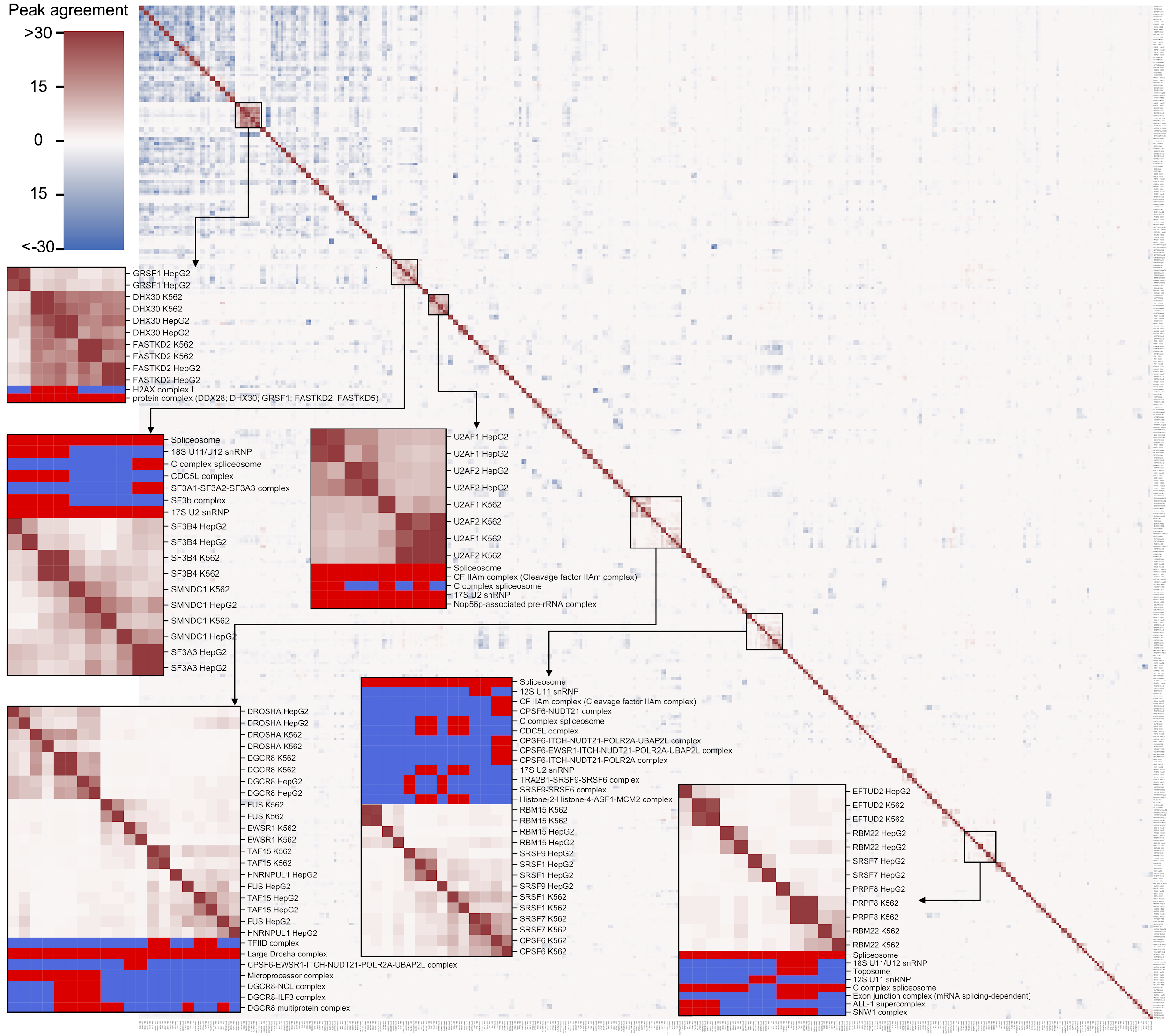
**Figure S2.** **Agreements of peaks identified between individual ENCODE samples.** The agreement is calculated as the Jaccard distance of the nucleotides in the peaks, where the intersection of two sets of peaks is the number of nts covered in both sets, while the union is the number of nts covered in at least one of the two sets. The color range is capped at a similarity of 0.4 to make the clusters more easily distinguishable. The top peaks are taken according to the FDR threshold (0.1), extending by 20 nts upstream and downstream from the crosslink site. Only samples with more than 100 peaks are included in this plot. The membership of RBPs in complexes is taken into account (based on CORUM [[74]](https://paperpile.com/c/tpj1xi/OVuA0)), by multiplying the value of the agreement by 1 if two proteins are known to participate in the same complex, and -1 otherwise. Resulting negative values are shown in blue, while positive values are shown in red. That is, shown in red are peak agreements of samples that either correspond to the same protein, or to proteins known to interact with each other in complexes. On the right, a few clusters of samples containing proteins that are known to take part in complexes are highlighted.


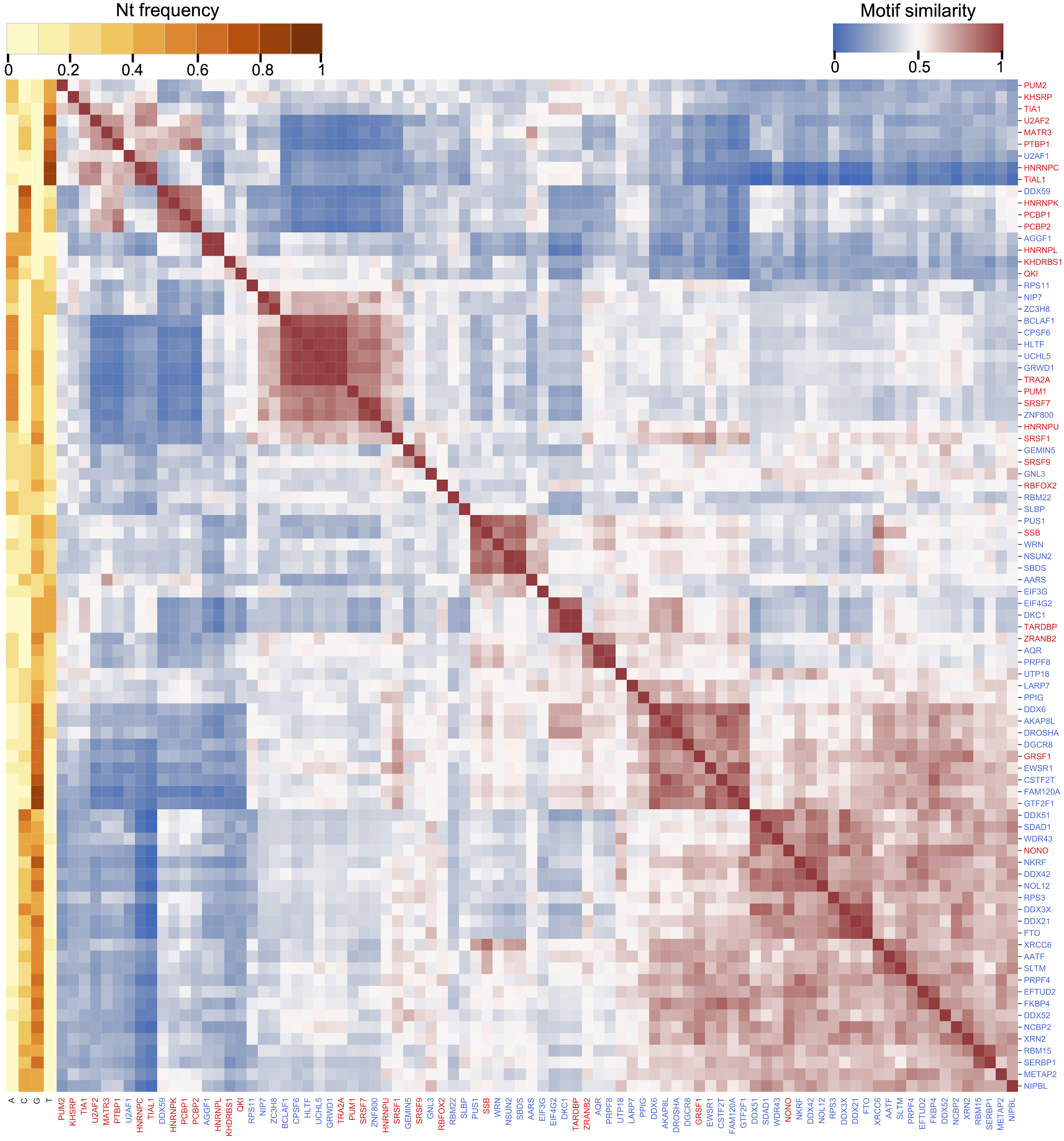


**Figure S3.** **Similarity of *de novo* predicted motifs of different RBPs.** Clustermap of *de novo* motif similarity of all RBPs covered in the ENCODE dataset. Similarity is calculated as described in the methods section (Calculation of motif similarity), taking for each RBP the motif that best explains all of the samples corresponding to that RBP. On the left, a representation of the nucleotide composition of the motif is shown, each column corresponding to one nucleotide, while the color indicates the relative frequency of that nucleotide averaged over all positions of the PWM. The RBP names are colored according to whether they have a motif (red) or not (blue) in ATtRACT.


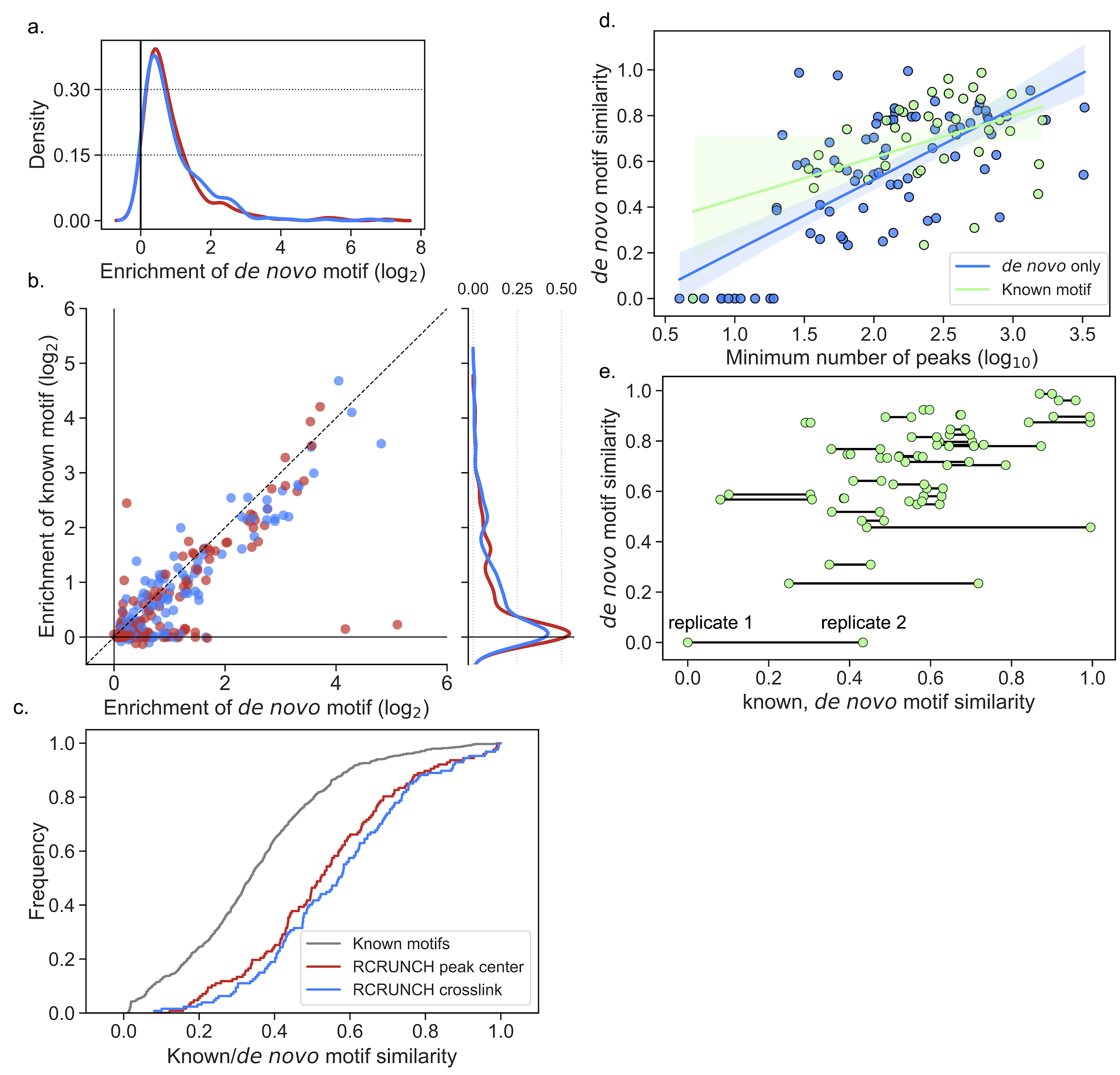


**Figure S4.** **RCRUNCH results for all ENCODE eCLIP data currently available.** **a.** Distribution of the enrichment scores of *de novo* motifs computed from peaks identified by RCRUNCH peak center (red) or RCRUNCH crosslink (blue). **b.** Scatter plot of the enrichment of the *de novo* motif predicted in the RCRUNCH-identified peaks from each sample, versus the enrichment score of the known motif for the protein assayed in the respective experiment. Marginal distributions of the known motif enrichments in RCRUNCH crosslink (blue) and RCRUNCH peak center (red) peaks are also shown. **c.** Cumulative density function of pairwise similarity scores for random pairs of known RBP-binding motifs (gray), known and *de novo* motifs identified from RCRUNCH crosslink sites (blue), known and de novo motifs identified for RCRUNCH peak center sites of individual RBPs (red). The same known motif was used for a given protein. **d.** Relationship between the similarity of *de novo* motifs inferred from replicate experiments and the minimum number of binding sites identified in these replicates. Experiments (each corresponding to an RBP and cell line) are colored according to whether (green) or not (blue) a motif was found in ATtRACT for the assayed RBP. **e.** Relationship between the similarity of *de novo* motifs identified in replicate experiments for a given RBP and the similarities of these *de novo* motifs and the known motif of the corresponding RBP. Lines connect replicate samples.


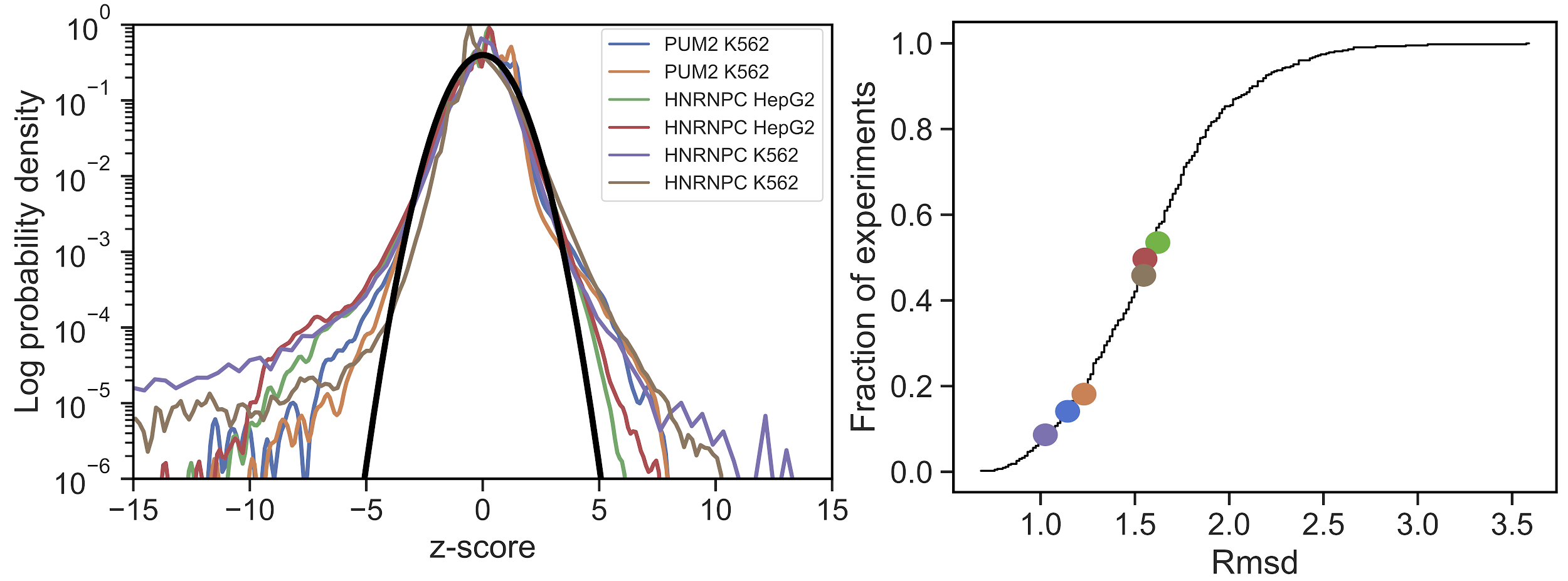


**Figure S5. Evaluation of the model used to identify enriched regions.** Left panel: z-score distributions for all the genomic windows with at least 2 reads in the IP samples for a random subset of samples (of benchmarked RBPs), compared to the expected distribution (in black). Right panel: cumulative distribution of root-square mean deviation of the empirical and predicted z-score distribution for all eCLIP samples in ENCODE. The samples shown in the left panel are highlighted with the same colors.


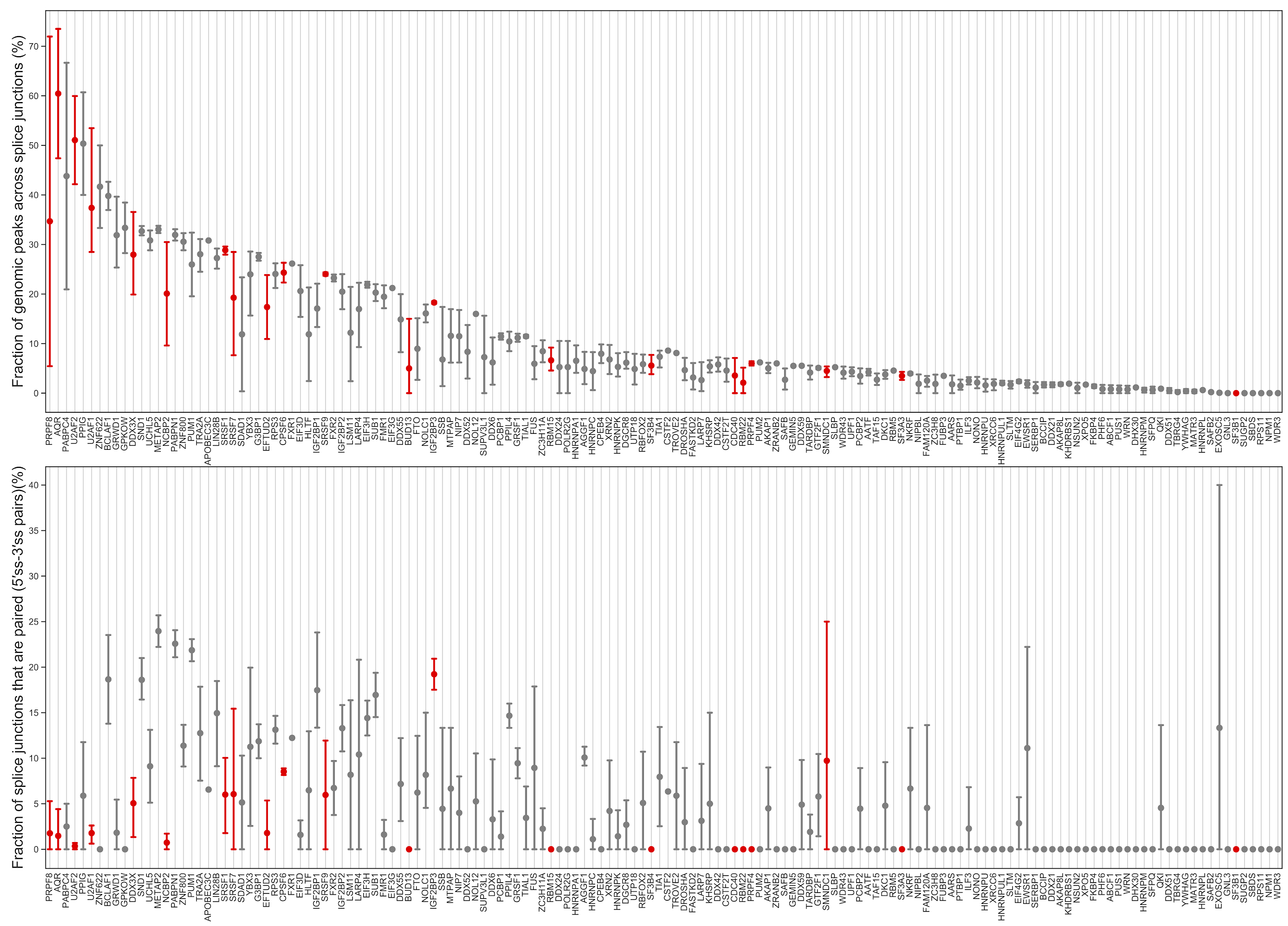


**Figure S6. Binding events spanning splice junctions. a.** Scatterplot of average fraction (and standard deviation) of peaks identified for the specified RBPs by RCRUNCH (genomic approach) that overlap with annotated splice junctions. The samples are ENCODE eCLIP samples and the average is calculated over replicates and cell lines. **b.** Scatterplot showing the fraction of the peaks that overlap a junction (e.g 3’ss) and also have a “pair” peak which overlaps the other side of the same junction (5’ss).

**
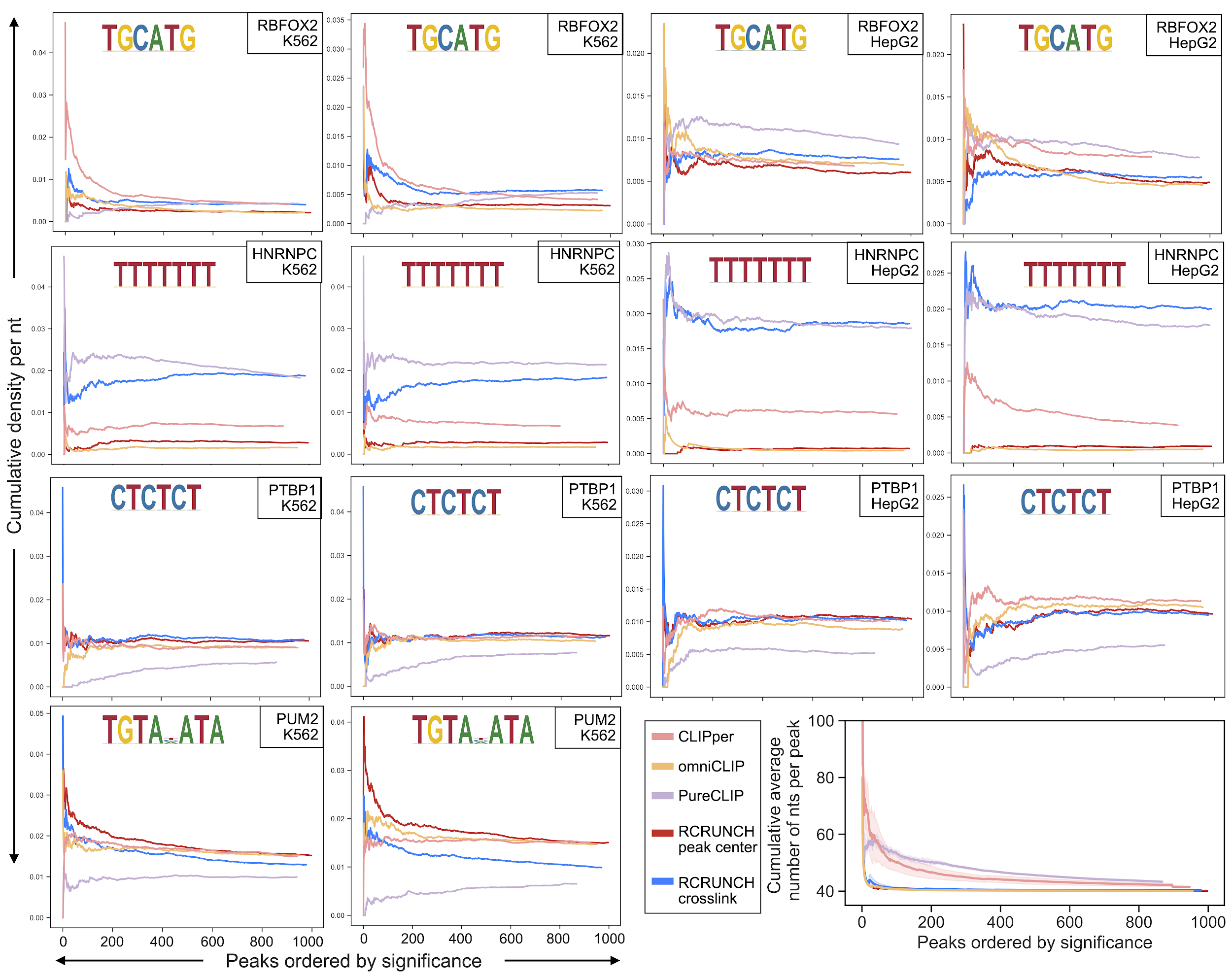
**

**Figure S7. Motif enrichments as a function of the number of top peaks.** Analyses were carried out for individual samples (IP vs. corresponding SMI) corresponding to one RBP and cell line. The top *x* peaks identified by a given tool, according to the tool’s significance measure, were used to calculate posterior probabilities of sites (matching known motifs from ATtRACT) with the MotEvo tool [[27]](https://paperpile.com/c/tpj1xi/j653Z) assuming uniform nucleotide frequencies and a prior probability for the background of 0.99. Sites with a posterior >= 0.3 per nucleotide in the peaks were used to calculate the average site density per nucleotide in peaks. The figures show that in general, the top peaks have the highest density of expected motifs. Bottom right panel: average number of nucleotides per peak as a function of the number of top peaks, sorted by significance. For each tool (indicated by the color), peaks were extracted from a given sample, overlapping peaks were merged and average of merged peak sizes over all benchmarked proteins and all samples were calculated. The standard deviations are also shown. The figure shows that CLIPper and PureCLIP tend to extract closely-spaced peaks, which are then merged to yield broader peaks.

.
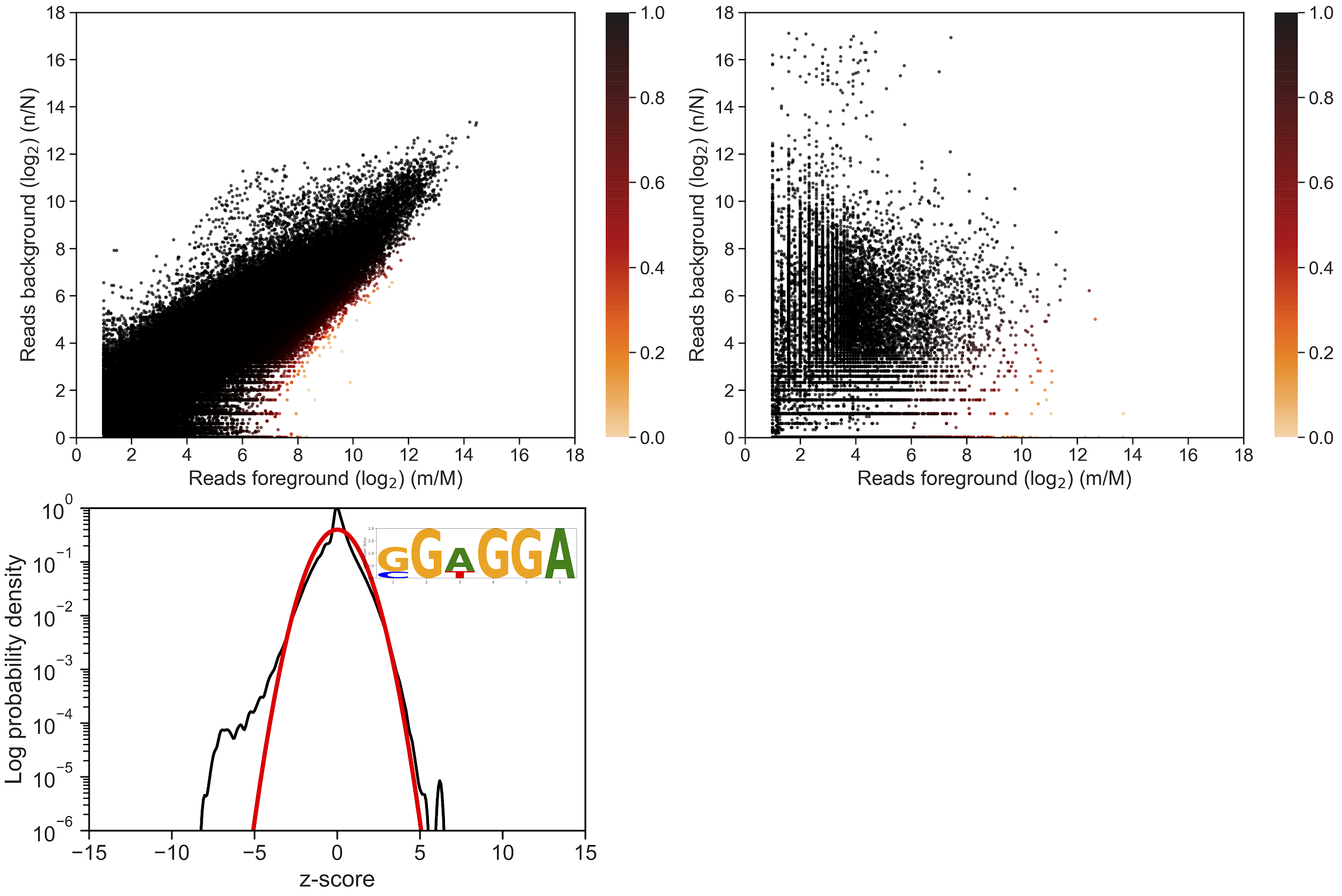


**Figure S8. RCRUNCH application to PAR-CLIP data.** RCRUNCH analysis was performed on two example datasets: for the fly CNBP protein [[75]](https://paperpile.com/c/tpj1xi/gg37), known to bind a GGA motif [[15,75]](https://paperpile.com/c/tpj1xi/gg37+znA3A), and the human PUM2 protein [[76]](https://paperpile.com/c/tpj1xi/GvVo), known to bind the UGUANAUA motif. The CNBP IP sample had a matched SMI control, whereas for the PUM2 PAR-CLIP we used as control RNA-seq data from [[76,77]](https://paperpile.com/c/tpj1xi/GvVo+CfnJ).
