# Supplementary material for: Improved analysis of (e)CLIP data with RCRUNCH yields a compendium of RNA-binding protein binding sites and motifs
Source: Genome Biol. 2023 Apr 17;24:77. doi: 10.1186/s13059-023-02913-0 (PMC10108518; doi:10.1186/s13059-023-02913-0)
Supplement: Supplementary file 7 — Additional file 7. Review_history. [file 13059_2023_2913_MOESM7_ESM.docx]

**1^st^ round**

**Reviewer 1**

Improved analysis of (e)CLIP data with RCRUNCH yields a compendium of RNA-binding protein binding sites and motifs
by Katsantoni et al.

Katsantoni and colleagues present the software RCRUNCH to simplify and streamline CLIP data processing from mapping to peak calling. The pipeline combines standard tools for next-generation sequencing data processing with a new peak calling algorithm and provides several extra features: RCRUNCH allows to include multi-mapping reads, offers the option to detect splice junction-spanning peaks and provides several modes of peak calling, e.g., taking into account complete reads or read starts (crosslinked positions). To test the software, the authors process ENCODE eCLIP data for four selected RNA-binding proteins (RBPs) in the K562 and HEPG2 cell lines, benchmark the results against three commonly used peak calling algorithms (CLIPper, omniCLIP, PureCLIP), and compare the obtained motifs with known consensus motifs. Lastly, the authors apply the pipeline to all available ENCODE eCLIP datasets and provide an overview of the identified motifs and the degree of overlap of binding sites between RBPs.

A detailed description of the transcriptome-wide RBP binding profiles is essential to understand gene expression regulation. A universal, adaptable and easy-to-use analysis pipeline for CLIP-type data as proposed here will be very valuable for the scientific community. However, several aspects should be revisited (see below).

The RCRUNCH tool is available as a Snakemake pipeline with clean encoding, a detailed documentation and a clear description of the config file required. The tool is parallelised and comes with all dependencies. Overall, it should be easy to use for everyone. The manuscript is very well written and accompanied by informative figures.

Major points:

1) The authors describe their approach as an automated analysis pipeline that can be used on different CLIP datasets. Yet, it appears that at present, the approach is only applicable to eCLIP data and only ever applied to those. For instance, the pipeline requires strand-specific paired-end sequencing data, which is specific for eCLIP (many CLIP datasets other than ENCODE eCLIP are sequenced with single-end reads), as well as a size-matched input (SMI) sample for the peak calling step. The authors should either re-write these parts of the manuscript or show the performance of RCRUNCH on at least one CLIP dataset from a protocol other than eCLIP.

2) The authors use the degree of peak overlap to compare the performance of different peak calling algorithms. I suspect that this measure could be strongly influenced by the total number of called peaks. Even when taking the top 1000 peaks, this set could represent the most confident peaks for one peak caller, while it may cover the complete peak set for another. This aspect should be taken into account for the benchmarking. Also, what is the length distribution of called peaks and how will this influence the overlap? How does library complexity (number of reads/unique reads/crosslinks per replicate/called peaks) relate to the measured performance of the peak callers?

The results of the benchmarking are presented in very condensed format that does not allow to evaluate what the underlying data looked like. It would be important to include genome browser views of peaks that were consistently detected by all tools compared to peaks that were specific to RCHRUNCH, but not found by the other tools.

3) The authors present two variants of RCRUNCH in which peak calling is based either on the read coverage centers (RCRUNCH peak center) or the most crosslinked positions (RCRUNCH crosslink). The comparisons of motif enrichment indicate that the obtained peaks are more accurate when calculated from crosslinked positions for some RBPs (RBFOX2, hnRNPC), but do not improve for others (PUM2, PTBP1). The authors attribute this observation to a different proportion of readthrough events for the different RBPs, which they support by a simulation of CLIP datasets with increasing readthrough frequencies.

The usage of crosslinking positions versus complete reads has been analysed before (PMID: 26260686, 28093074). Both publications provide measures to test for the contribution of reverse transcription abortion and readthrough and provide possible explanations for the observations made. These results should be taken into account here.

When using the read coverage centers instead of crosslinked positions, the obtained peak locations are shifted downstream as a function of the read length. Differences in fragment lengths could influence the differences in distances between peak centers and motifs that are observed for the different RBPs. The authors should include read length as a factor into this analysis and also report the average read length per RBP (after adapter trimming of individual reads/ estimated fragment length from paired-end).

Given the authors' observation that RCRUNCH crosslink improved the peak detection for some RBPs and did not generally impair the others, this mode could be suggested as the default approach in the tool. Also, are there any features that a user could check to choose whether to run RCRUNCH peak center or RCRUNCH crosslink on a given dataset?

4) A selling point of RCRUNCH is that it provides additional features including the use of the correct use of junction-spanning reads which will split up the signal into multiple distant peaks. To correct for this, the authors determine a representative isoform for each gene which is most abundant in the foreground sample according to Salmon. This is an elegant approach. Conceptually, it would be good to ensure that Salmon can be used on CLIP data (given the incomplete coverage compared to real RNA-seq data). This could be tested by comparing reconstructed transcript isoforms from RNA-seq data and CLIP data for a cytoplasmatic RBP from the same cell line. More generally, it should be noted that this issue only arises when using complete reads (RCRUNCH peak center), but will be circumvented automatically for most RBPs when focusing on the crosslinked positions.

5) In the final step, the authors apply RCRUNCH to all available ENCODE data to construct "a comprehensive and homogeneous resource of in vivo-bound RBP sequence motifs". However, it is unclear if and how the results of these analyses are available to the users.

The obtained peak locations and the derived motifs should be made available for all processed ENCODE eCLIP data.

How do the obtained motifs fit to the information in ATtRACT? Similarly, how do the results compare to the original analysis of the ENCODE eCLIP data (PMID: 32728246)?

6) To enable the user to evaluate the results and also to decide whether the correct RCRUNCH mode was used on the analysed dataset, it will be important to output not only peaks, but also quality measures that inform on the reliability of the obtained information.


Minor points:

1) Tick marks are missing on the x-axes of several plots.

2) Figure 1c. Dark grey distribution of the background read coverage is not visible.

3) hnRNPC binding on Alu elements was reported in PMID: 23374342.

4) SMI: abbreviation not introduced.

**Reviewer 2**

The authors provide an interesting method to analyse single-nucleotide resolution CLIP data for detection of RNA-binding protein binding sites, being able to make use of uniquely and multimapped reads. The manuscript is well written and the method interesting for the community, especially, because the analysis of eCLIP data is full of challenges. I would like to raise some questions and suggestions to improve the quality of the manuscript.

1) The CRUNCH model was extensively validated on ChIP data. CLIP data inherently has different properties and binding modes and patterns of RNA-binding proteins are different to transcription factors binding DNA. Can you please discuss why CRUNCH is applicable to CLIP data, limitations etc.

2) The Authors have addressed RBPs with sequence motifs in the manuscript. How does RCRUNCH perform for identifying peaks for RBPs binding to RNA secondary structures, such as the histone stem loop binding protein SLBP?

3) The identification of broad genomic regions whose coverage by reads is significantly higher in the foreground compared to the background (related to Fig 1b, 1c) raises a couple of questions: How are samples normalized, what is the statistical test? How is a removal of certain RNA types justified? Why do you not take advantage of replicate information for calling those regions? How robust is RCRUNCH to different sequencing depth, e.g. if the SMI has way higher or lower coverage?

4) Page 7 line 38 onwards and Page 28 section Calculation of peak agreement between replicate samples and between methods: The agreement between peaks across replicates are computed using Jaccard distance. But, how do the z-scores of overlapping peaks across replicates compare?

5) Figure 2b: Arguably, the measure of "maximum agreement" should be replaced by a robust measure. Can you please show a distribution of Jaccard indices (maybe as violin/boxplot)? Alternatively a median would help to describe a general improvement.

6) Is RCRUNCH suitable for eCLIP data from RBPs with multiple motifs?

7) The manuscript is based on bioinformatic de-novo motif discovery and comparing to motifs from ATtRACT database. Are there any other datasets like RNA Bind'n'Seq to cross validate, like used in https://academic.oup.com/bib/article/22/6/bbab149/6278600?

8) Page 7 lines 14-16: Please clarify whether the authors have used clipper results from the original study (2016), with or without the 50' nt extension, or the revised set of results (2020).
How do the performance scores on Figure 2 panels a-c appear upon comparison with original or revised set?

9) Please discuss the choice for using MetEvo for de novo motif prediction. Is the tool also benchmarked for RBP binding motifs? Is MotEvo comparable to STREME from the MEME suite?

10) page 13 etc. The finding of Alu elements is not surprising, please add "Direct Competition between hnRNP C and U2AF65 Protects the Transcriptome from the Exonization of Alu Elements" Zarnack K. et al. 2013. This could be incorporated as validation.

11) Page 25 lines 42 - 48: Since the technologies behind ChIP-seq and CLIP - sequencings are different, please provide additional comments on how transferable are the observations about noise levels from ChIP datasets to CLIP datasets.

12) How does RCRUNCH automate the reproducible analysis of CLIP data - please discuss
Minor comments:
- Title: RNAbinding to "RNA-binding"
- page 1, line 7: add Castello 2012, Baltz 2012 references for RNA Interactome capture
- page 1, line 15: like RIC have uncovered proteins -> many proteins. (Castello et al, Molecular Cell 2016 has identified that more than half of the binding occurs in disordered regions).
- page 1, line 22: add Gebauer et al. RNA-binding proteins in human genetic
Disease. Nature Rev. Genetics 2021 reference
- page 2, line 37 replace "-" with ","
- page 3, line 12: "it is less clear" is not an appropriate claim. To date, it is clear that CLIP background is more complex than ChIP-seq due to crosslink effects (eCLIP, van Nostrand 2016). One does know that size-matched input controls or Complementary Controls (multiple other IPed non-size selected samples, see also Lorenz et al 2022 "Multiplexed transcriptome discovery of RNA binding protein binding sites by antibody-barcode eCLIP" bioRxiv) are appropriate for i/eCLIP, and that RNAseq and other forms are not. The lack of appropriate controls in the beginning of iCLIP practically was based on wrong assumptions that CLIP-seq is analogous to ChIP-seq. I would suggest rephrasing this.
- page 3 line 26-27: missing reference, RNA-seq normalization
- page 4, line 12:14. To the best of my knowledge, pureCLIP does not take advantage of the crosslink-induced mutations.
- line: 26 followed by high-throughput sequencing
- Page 25 line 14: Authors define selection strategy as:
AS score transcriptome alignment > AS score genome alignment - 3. But, the rationale behind using - 3 is not explained.
- page 26, lines 26-41: Please state FDR and z-score thresholds used in analysis in the main manuscript.
- Figure 1 panel c: CLIP read coverage (light gray) vs background read coverage (dark gray) is not easy to differentiate. Please think about an alternate color scheme.
- Figure 1b: please use eCLIP when describing eCLIP (ENCODE) data. Please use IP for samples, SMI for controls. The abbreviation CLIP is describing a method. (for the whole manuscript)
- Figure 2 panel c: It is not clear how the information on the heatmap is represented.
- Figure 3 panel a: please add de-novo sequence motifs identified from omniCLIP and PureCLIP peaks as well.
- Page 7 line 44: The agreement of the top peaks…. Please indicate the number of peaks selected here.
- Page 18 Figure panel c and lines 54-55: Please make sure that the scale of the color panel range (0-30) and the figure legend (The color range is capped at a similarity of 0.4….) are the same.
- whole manuscript: please use subscript for 2 in log2
- whole manuscript: for consistency in the literature, please spell 'clipper' 'CLIPper'
- formatting consistency: Figure S1 Legend.
- Figure 5 panels c, f and i: Are PTBP1 and PUM2 data points missing in these panels? Are they hidden behind other data points? If so, please adjust the transparency so that these are also visible.
- In Figure 1b, why was replicated 2 used?
- Figure 2c : Please label the methods which they are compared to on the right side

**Authors’ response**

Reviewer 1

Improved analysis of (e)CLIP data with RCRUNCH yields a compendium of RNA-binding protein binding sites and motifs by Katsantoni et al.

Katsantoni and colleagues present the software RCRUNCH to simplify and streamline CLIP data processing from mapping to peak calling. The pipeline combines standard tools for next-generation sequencing data processing with a new peak calling algorithm and provides several extra features: RCRUNCH allows to include multi-mapping reads, offers the option to detect splice junction-spanning peaks and provides several modes of peak calling, e.g., taking into account complete reads or read starts (crosslinked positions). To test the software, the authors process ENCODE eCLIP data for four selected RNA-binding proteins (RBPs) in the K562 and HEPG2 cell lines, benchmark the results against three commonly used peak calling algorithms (CLIPper, omniCLIP, PureCLIP), and compare the obtained motifs with known consensus motifs. Lastly, the authors apply the pipeline to all available ENCODE eCLIP datasets and provide an overview of the identified motifs and the degree of overlap of binding sites between RBPs.

A detailed description of the transcriptome-wide RBP binding profiles is essential to understand gene expression regulation. A universal, adaptable and easy-to-use analysis pipeline for CLIP-type data as proposed here will be very valuable for the scientific community. However, several aspects should be revisited (see below).

The RCRUNCH tool is available as a Snakemake pipeline with clean encoding, a detailed documentation and a clear description of the config file required. The tool is parallelised and comes with all dependencies. Overall, it should be easy to use for everyone. The manuscript is very well written and accompanied by informative figures.

We thank the reviewer for emphasizing the quality of our software.

Major points:

1) The authors describe their approach as an automated analysis pipeline that can be used on different CLIP datasets. Yet, it appears that at present, the approach is only applicable to eCLIP data and only ever applied to those. For instance, the pipeline requires strand-specific paired-end sequencing data, which is specific for eCLIP (many CLIP datasets other than ENCODE eCLIP are sequenced with single-end reads), as well as a size-matched input (SMI) sample for the peak calling step. The authors should either re-write these parts of the manuscript or show the performance of RCRUNCH on at least one CLIP dataset from a protocol other than eCLIP.

We thank the reviewer for these suggestions, which we have implemented as follows.

To extend the scope of RCRUNCH, we are now providing a single-end read-based implementation as well. With regards to other types of CLIP, the main issue, already discussed in our manuscript, is that while there is substantial noise in CLIP data, appropriate background samples that enable estimation of this noise are not generally produced. Moreover, there are no datasets generated with different protocols in the same system, so that the performance of the different techniques can be compared. For these reasons, as well as because eCLIP data is by far the most extensive data type available, we focused our efforts on analyzing eCLIP data. Nevertheless, to address the reviewer’s request, we searched for CLIP datasets with appropriate background in public repositories and we indeed found a PAR-CLIP dataset for the fly CNBP protein, also analyzed in (Drewe-Boss et al. 2018) et al. The RCRUNCH results for this data set (Fig. S8) show that we obtain the expected sequence motif. Thus, when appropriate background samples are available, RCRUNCH can identify relevant sites and motifs, even when the sample preparation was done according to protocols other than eCLIP. On the contrary, for a PUM2 PAR-CLIP data for which only RNA-seq background was available, the RCRUNCH model does not apply and we could not identify meaningful peaks. We have revised the discussion section of our manuscript to emphasize this point.

2) The authors use the degree of peak overlap to compare the performance of different peak calling algorithms. I suspect that this measure could be strongly influenced by the total number of called peaks. Even when taking the top 1000 peaks, this set could represent the most confident peaks for one peak caller, while it may cover the complete peak set for another. This aspect should be taken into account for the benchmarking.

The reviewer is right in pointing out that tools can differ widely in the number of peaks that they report, and we did have this aspect in mind when comparing the tools. It is somewhat challenging to summarize all these results for all methods, all proteins and all cutoffs on peak score. Nevertheless, to address the reviewer’s comment we now present in Fig. S7 summaries of site density as a function of the number of top peaks considered for each method. The results show that the performances that we reported in Fig. 2 for the top 1000 sites are strongly consistent across the same range of top peak numbers. Specifically, while other methods have highly variable performance, depending on the RBP, RCRUNCH gives reliable results across all proteins, with the top sites showing the strongest motif enrichments.

Also, what is the length distribution of called peaks and how will this influence the overlap?

Again the reviewer points out an important issue: the definition of a peak can and does differ between methods. However, most methods really aim to reach single nucleotide resolution, and therefore, for our comparisons we extracted peaks of the same size, centered on the position that each method considered the most likely crosslinked position within a coverage peak. Only CLIPper reported broader regions and in this case we took the center of these regions as the reference crosslink position.

We chose a peak size of 40 nucleotides, close to the fragment size. We finally clustered overlapping 40-nucleotide regions. As shown in Fig. S7, the top PureCLIP (and to some extent CLIPper) peaks overlap with each other, because these tools report very closely spaced positions as crosslinked, and the sites that we then extract for these tools overlap and are merged. In any case, in the tests that we did we factored out the length by calculating site density (Fig. S7).

How does library complexity (number of reads/unique reads/crosslinks per replicate/called peaks) relate to the measured performance of the peak callers?

As the reviewer surely appreciates, all methods were benchmarked on the same data sets and we reported the performance of each method on each data set. In principle, these library statistics depend on a variety of parameters, including the number of binding sites and distribution of affinities they have for the RBP. In the newly inserted Supplementary Table 2 we report library statistics for all RPBs including those that we benchmarked. We did not see a consistent pattern of peak caller performance with these statistics but of course, it is not possible to draw quantitative conclusions based on a small number of proteins for which the “ground truth” is reasonably known.

The results of the benchmarking are presented in very condensed format that does not allow to evaluate what the underlying data looked like. It would be important to include genome browser views of peaks that were consistently detected by all tools compared to peaks that were specific to RCRUNCH, but not found by the other tools.

We have added Supplementary table 1c to show which of the RCRUNCH peaks overlap with peaks from the other tools. The underlying data is of course the same, and it was difficult to pick specific examples of peaks that were picked by different combinations of tools.

3) The authors present two variants of RCRUNCH in which peak calling is based either on the read coverage centers (RCRUNCH peak center) or the most crosslinked positions (RCRUNCH crosslink).

The comparisons of motif enrichment indicate that the obtained peaks are more accurate when calculated from crosslinked positions for some RBPs (RBFOX2, hnRNPC), but do not improve for others (PUM2, PTBP1). The authors attribute this observation to a different proportion of readthrough events for the different RBPs, which they support by a simulation of CLIP datasets with increasing readthrough frequencies. The usage of crosslinking positions versus complete reads has been analysed before (PMID: 26260686, 28093074). Both publications provide measures to test for the contribution of reverse transcription abortion and readthrough and provide possible explanations for the observations made. These results should be taken into account here.

We thank the reviewer for pointing out these articles, which we have now mentioned in the section “RCRUNCH helps elucidate how RBPs interact with and crosslink to RNAs” on the use of crosslink positions vs. peak centers. Indeed, to what extent the reverse transcriptase aborts at crosslink sites has been an open question for quite some time, influenced by all factors that affect the crosslinking efficiency and configuration of crosslinked nucleotides. A model that takes into account all of these factors to interpret the pattern of read distribution is very challenging to construct and beyond the scope of our study, as it would imply constructing a completely different model. Our focus was on a general model for peak extraction based on the read coverage, which should apply for different fragment lengths or probabilities of readthrough, even though it may not provide nucleotide resolution. We further provide the position of most frequent truncation within each peak and use the de novo motif enrichment as an indication of the positional accuracy of binding site identification. We think that our study and approach contribute to the discussion about the frequency of readthrough in CLIP data in a number of ways, i.e. by providing a general method that performs well on average, for RBPs with widely different binding motifs and crosslinking preferences, and also through the simulation framework that can be further developed to interpret the experimental data.

When using the read coverage centers instead of crosslinked positions, the obtained peak locations are shifted downstream as a function of the read length. Differences in fragment lengths could influence the differences in distances between peak centers and motifs that are observed for the different RBPs. The authors should include read length as a factor into this analysis and also report the average read length per RBP (after adapter trimming of individual reads/ estimated fragment length from paired-end).

As requested by the reviewer we have generated the distributions of fragments lengths for the ENCODE eCLIP libraries based on the paired-end read mappings and found this to be 60-83 nucleotides, with 73% of the samples in the range of 70-80 nucleotides (Suppl. Table 2). Thus, the variation in fragment length is generally small. This was also the case for the samples for benchmarked RBPs.

Given the authors' observation that RCRUNCH crosslink improved the peak detection for some RBPs and did not generally impair the others, this mode could be suggested as the default approach in the tool. Also, are there any features that a user could check to choose whether to run RCRUNCH peak center or RCRUNCH crosslink on a given dataset?

RCRUNCH runs by default the two modes and we use the enrichment of the de novo inferred motifs as a measure for which of the two ways of identifying binding sites comes closer to site resolution. We added a few words to clarify this in the discussion.

4) A selling point of RCRUNCH is that it provides additional features including the use of the correct use of junction-spanning reads which will split up the signal into multiple distant peaks. To correct for this, the authors determine a representative isoform for each gene which is most abundant in the foreground sample according to Salmon. This is an elegant approach. Conceptually, it would be good to ensure that Salmon can be used on CLIP data (given the incomplete coverage compared to real RNA-seq data). This could be tested by comparing reconstructed transcript isoforms from RNA-seq data and CLIP data for a cytoplasmatic RBP from the same cell line.

Indeed, picking up the appropriate isoform is challenging, because the CLIP data could be very sparse. On the other hand, the abundance in RNA-seq is not necessarily predictive of the probability of interaction with the RBP. This is perhaps clearest for binding sites that occur in alternatively-spliced exons. Of course, CLIP sample preparation is also quite different compared to RNA-seq sample preparation. The main reason for the approach that we chose is that we need to ensure that the transcript regions that are apparent in CLIP are also assessed from the background sample. Like with the other questions about an appropriate background, this aspect could be addressed deeper in future work. More generally, it should be noted that this issue only arises when using complete reads (RCRUNCH peak center), but will be circumvented automatically for most RBPs when focusing on the crosslinked positions. With an analysis that solely uses read starts the problem would be mitigated. However, we do not think that it will be completely eliminated, for a number of reasons. First, the read starts are not always the most informative of the location of the sites, as the reviewer was pointing out above. Then, reads that map across splice junctions may be missed or incorrectly reported (especially if the user does not use splicing-aware aligners). When the reads are not lost in the mapping, the binding sites may still be split across splice junctions and the extraction of the sequence for motif finding may be incorrect. In addition, sites that span splice junctions may result in duplicate genomic peaks (one in the upstream and the other in the downstream exon), with further complications for the downstream analysis. Thus, we think that even when using crosslink positions, it is beneficial to increase the number of reads that are accurately mapped.

5) In the final step, the authors apply RCRUNCH to all available ENCODE data to construct "a comprehensive and homogeneous resource of in vivo-bound RBP sequence motifs". However, it is unclear if and how the results of these analyses are available to the users. The obtained peak locations and the derived motifs should be made available for all processed ENCODE eCLIP data.

We provide the inferred motifs as Supplementary Table 3. Furthermore, we have uploaded all of our results to zenodo, a data repository that is guaranteed to be maintained indefinitely. We included the link to this repository in the Availability of Data and Materials section.

How do the obtained motifs fit to the information in ATtRACT? Similarly, how do the results compare to the original analysis of the ENCODE eCLIP data (PMID: 32728246)?

The comparison with ATtRACT motifs is shown in Fig. S4 of our manuscript. We could not do a similar analysis of ENCODE motifs because we could not obtain the PWMs (the ENCODE analysis is based on kmers, which are then clustered to derive PWMs).

6) To enable the user to evaluate the results and also to decide whether the correct RCRUNCH mode was used on the analysed dataset, it will be important to output not only peaks, but also quality measures that inform on the reliability of the obtained information.

We are not sure what the reviewer would like us to add. For each peak we provide the z-score of read enrichment. Moreover, for each data set we provide the de novo motifs with their enrichment score to help the user assess whether the coverage or the read starts are more informative for the RBP binding motif.

Minor points:

1) Tick marks are missing on the x-axes of several plots.

We thank the reviewer for catching these, we have added the tick marks.

2) Figure 1c. Dark grey distribution of the background read coverage is not visible.

We clarified in the legend of the figure that the envelope of this background coverage is shown with yellow.

3) hnRNPC binding on Alu elements was reported in PMID: 23374342.

We thank the reviewer for catching this, we have included this reference.

4) SMI: abbreviation not introduced.

We have corrected this omission.

Reviewer #2: The authors provide an interesting method to analyse single-nucleotide resolution CLIP data for detection of RNA-binding protein binding sites, being able to make use of uniquely and multimapped reads. The manuscript is well written and the method interesting for the community, especially, because the analysis of eCLIP data is full of challenges. I would like to raise some questions and suggestions to improve the quality of the manuscript.

1) The CRUNCH model was extensively validated on ChIP data. CLIP data inherently has different properties and binding modes and patterns of RNA-binding proteins are different to transcription factors binding DNA. Can you please discuss why CRUNCH is applicable to CLIP data, limitations etc.

The reviewer rightly notes that there are some differences between ChIP and CLIP, so that CRUNCH may not be directly applicable to CLIP data. A main difference is in fact in the background sample. The background coverage in ChIP is sparse and more uniform than the background coverage in CLIP. This is why CRUNCH uses broader genomic windows for estimating the coverage from the background sample compared to the foreground sample. We found that for CLIP, we obtain better results if we use similar size windows for foreground and background, smaller than those used in CRUNCH. The goodness of fit of empirical z-score distributions with respect to the standard normal (shown if Fig. S5) are comparable to those obtained in CRUNCH.

2) The Authors have addressed RBPs with sequence motifs in the manuscript. How does RCRUNCH perform for identifying peaks for RBPs binding to RNA secondary structures, such as the histone stem loop binding protein SLBP?

The peak finding precedes the motif analysis. We checked specifically the peaks for SLBP and found that we obtained 162 and 307 peaks in two replicate samples. 140 and 187 of these are located in histone transcripts (“structural constituent of chromatin” Gene Ontology annotation). It is possible that some of the RBPs for which we did not find a specific sequence motif bind secondary structure elements. However, the number of known examples is small, and we did not implement a search for secondary structure elements in RCRUNCH.

3) The identification of broad genomic regions whose coverage by reads is significantly higher in the foreground compared to the background (related to Fig 1b, 1c) raises a couple of questions: How are samples normalized, what is the statistical test? How is a removal of certain RNA types justified? Why do you not take advantage of replicate information for calling those regions? How robust is RCRUNCH to different sequencing depth, e.g. if the SMI has way higher or lower coverage?

The model we used to detect regions with increased coverage in foreground compared to background is given in section “The RCRUNCH model for the detection of RBP binding regions”. We do not remove certain types of RNAs by default, but we provide this as an option, as it is a strategy often used in the field. We here focused on extracting the most likely sites from each sample and used the replicates to demonstrate the reproducibility of the method’s results. The user always has the opportunity to combine multiple replicates to filter the sites according to their criteria of interest. While we did not explore the sensitivity of RCRUNCH to sequencing depth, we now provide the sample sizes in Supplementary Table 2.

4) Page 7 line 38 onwards and Page 28 section Calculation of peak agreement between replicate samples and between methods: The agreement between peaks across replicates are computed using Jaccard distance. But, how do the z-scores of overlapping peaks across replicates compare?

Variability in the degree of enrichment within individual windows between replicates is a well-known issue in CLIP experiments (Hafner et al. 2021). In light of this, we wanted to use a more robust and generally applicable method of a tool’s reproducibility. In the new panel b of Fig. 2 we now show the distribution of Jaccard indices of peaks extracted by different tools from replicate samples. As the reviewer can appreciate, the peak overlap between replicates is not very high, especially for tools other than RCRUNCH.

5) Figure 2b: Arguably, the measure of "maximum agreement" should be replaced by a robust measure. Can you please show a distribution of Jaccard indices (maybe as violin/boxplot)? Alternatively a median would help to describe a general improvement.

We thank the reviewer for this suggestion, we have replaced panel b with one showing the distributions of Jaccard indices of peak overlaps computed from replicate samples by each of the tools as violin plots.

6) Is RCRUNCH suitable for eCLIP data from RBPs with multiple motifs?

The peak finding and motif finding are separate steps in RCRUNCH. Thus, the user is able to first identify the coverage peaks and then search for possibly more than one motif that may be enriched in these peaks. In fact, in our default setting we search for two motifs and we report the one that has the overall highest enrichment across multiple runs of the Phylogibbs algorithm.

7) The manuscript is based on bioinformatic de-novo motif discovery and comparing to motifs from ATtRACT database. Are there any other datasets like RNA Bind'n'Seq to cross validate, like used in https://academic.oup.com/bib/article/22/6/bbab149/6278600?

We chose ATtRACT because it contains models (in particular positional weight matrices, not k-mer scores) obtained with a variety of in vivo and in vitro methods, including some of the methods covered in the paper pointed out by the reviewer. In fact, for the benchmark we tested all models for a given RBP that were present in ATtRACT and we reported the one that had the highest signal in the CLIP peaks.

8) Page 7 lines 14-16: Please clarify whether the authors have used clipper results from the original study (2016), with or without the 50' nt extension, or the revised set of results (2020).

How do the performance scores on Figure 2 panels a-c appear upon comparison with original or revised set?

We have used the peaks files from the ENCODE repository, which is the official portal for these data. The files have been generated after the original 2016 publication.

9) Please discuss the choice for using MetEvo for de novo motif prediction. Is the tool also benchmarked for RBP binding motifs? Is MotEvo comparable to STREME from the MEME suite?

We do not use MotEvo for de novo motif prediction. We use PhyloGibbs for de novo motif finding (PhyloGibbs is a Gibbs sampling motif finder that is highly versatile, i.e. allowing to search for multiple motifs in parallel, with variable numbers of motifs per region, and allowing strand-specific motifs as is appropriate for RBP motifs). We use MotEvo only to predict binding sites of the motifs and its results are used to score enrichment using a likelihood model. We use MotEvo mainly because it is a rigorous Bayesian algorithm that provides posterior probabilities for each of its predicted sites, which is used in our likelihood enrichment score. Note that this combination of PhyloGibbs/MotEvo was used in the CRUNCH paper (Berger et al. 2019) and extensive benchmarking on ChIP-seq data confirmed the excellent performance of this motif finding approach. Our PhyloGibbs/MotEvo combination is rather different from STREME, which is a de novo motif finder that uses a Fisher exact test (or Binomial test) statistics to first find enriched exact words, then approximate word matches, and then iteratively build enriched motifs from those. It is specifically designed for large datasets and the authors of STREME in fact suggest to use the standard MEME motif finding for smaller datasets.

Regarding the specific benchmarking for RBP motifs, we are not aware of any fundamental differences between motif finding for TF binding motifs and RBP motifs (other than the obvious strand-specificity, of course, which we enforce in the RCRUNCH workflow). Indeed as far as we know the PhyloGibbs, MotEvo, STREME and MEME methods have all been mainly benchmarked on ChIP-seq data for TFs and MEME has been routinely used for motif finding on CLIP data.

Finally, our results show directly that for the cases when the motifs are known, they are well reproduced by our approach.

10) page 13 etc. The finding of Alu elements is not surprising, please add "Direct Competition between hnRNP C and U2AF65 Protects the Transcriptome from the Exonization of Alu Elements" Zarnack K. et al. 2013. This could be incorporated as validation.

Indeed, we had an incorrect citation at the respective place in the manuscript. We have now corrected it and the reference to the Alu element study is included.

11) Page 25 lines 42 - 48: Since the technologies behind ChIP-seq and CLIP - sequencings are different, please provide additional comments on how transferable are the observations about noise levels from ChIP datasets to CLIP datasets.

The main assumption of the noise model of both CRUNCH and RCRUNCH is that the observed read count within a given region is subject to a combination of multiplicative noise (from technical noise in such steps as fragmentation and PCR amplification, leading to log-normal fluctuations in read density) and Poisson sampling noise from the sequencing itself. This is a quite general model and we propose that it is applicable to both ChIP-seq and CLIP data, in spite of the substantial differences between these two sequencing methods. Importantly, the parameters of this noise model are fitted to the observations of each dataset and RCRUNCH provides results that allow users to specifically verify to what extent the fitted noise model fits the observed read-count fluctuations (see also newly added Fig. S5).

12) How does RCRUNCH automate the reproducible analysis of CLIP data - please discuss

RCRUNCH is implemented in the Snakemake workflow language. The user only needs to provide a configuration file, after which the data analysis is done automatically, from reads to sites and motifs, without requiring user intervention. Individual components are containerized (docker) for version control and the workflow and configuration files have been deposited in github (link in the Availability of Data and Materials section).

Minor comments:

- Title: RNAbinding to "RNA-binding"

This seems to have been a mistake in the file format conversion.

- page 1, line 7: add Castello 2012, Baltz 2012 references for RNA Interactome capture

We replaced the reference to the review with references to these original work.

- page 1, line 15: like RIC have uncovered proteins -> many proteins. (Castello et al, Molecular Cell 2016 has identified that more than half of the binding occurs in disordered regions).

- page 1, line 22: add Gebauer et al. RNA-binding proteins in human genetic Disease. Nature Rev. Genetics 2021 reference

We have added this reference as well.

- page 2, line 37 replace "-" with ","

Fixed.

- page 3, line 12: "it is less clear" is not an appropriate claim. To date, it is clear that CLIP background is more complex than ChIP-seq due to crosslink effects (eCLIP, van Nostrand 2016). One does know that size-matched input controls or Complementary Controls (multiple other IPed non-size selected samples, see also Lorenz et al 2022 "Multiplexed transcriptome discovery of RNA binding protein binding sites by antibody-barcode eCLIP" bioRxiv) are appropriate for i/eCLIP, and that RNAseq and other forms are not. The lack of appropriate controls in the beginning of iCLIP practically was based on wrong assumptions that CLIP-seq is analogous to ChIP-seq. I would suggest rephrasing this.

We have rephrased the sentence. We fully agree with the reviewer that SMI works for i/eCLIP, and we further emphasize this in the comparison with RNA-seq.

- page 3 line 26-27: missing reference, RNA-seq normalization

We added a representative reference.

- page 4, line 12:14. To the best of my knowledge, pureCLIP does not take advantage of the crosslink-induced mutations.

Thank you for catching this, we meant more generally crosslinking-induced events (truncations for PureCLIP). We corrected this.

- line: 26 followed by high-throughput sequencing

We cannot find the mistake the reviewer is referring to here.

- Page 25 line 14: Authors define selection strategy as:

AS score transcriptome alignment > AS score genome alignment - 3. But, the rationale behind using - 3 is not explained.

The value of -3 is arbitrary, we simply wanted to favor alignments to transcriptome (as we specify in the text).

- page 26, lines 26-41: Please state FDR and z-score thresholds used in analysis in the main manuscript.

We have added this information in the Methods section.

- Figure 1 panel c: CLIP read coverage (light gray) vs background read coverage (dark gray) is not easy to differentiate. Please think about an alternate color scheme.

We have changed the color of the background to yellow.

- Figure 1b: please use eCLIP when describing eCLIP (ENCODE) data. Please use IP for samples,

SMI for controls. The abbreviation CLIP is describing a method. (for the whole manuscript)

- Figure 2 panel c: It is not clear how the information on the heatmap is represented.

We specified this in the legend so as to not overload the figure panel.

- Figure 3 panel a: please add de-novo sequence motifs identified from omniCLIP and PureCLIP peaks as well.

This would mean carrying out motif analyses for the peaks provide by other methods, which is not the scope of our study.

- Page 7 line 44: The agreement of the top peaks.... Please indicate the number of peaks selected here.

We added this information.

- Page 18 Figure panel c and lines 54-55: Please make sure that the scale of the color panel range (0-30) and the figure legend (The color range is capped at a similarity of 0.4....) are the same.

Thank you for catching this. Fixed.

- whole manuscript: please use subscript for 2 in log2

- whole manuscript: for consistency in the literature, please spell 'clipper' 'CLIPper'

We have fixed this as well.

- formatting consistency: Figure S1 Legend.

Thank you for picking this up, we fixed it.

- Figure 5 panels c, f and i: Are PTBP1 and PUM2 data points missing in these panels? Are they hidden behind other data points? If so, please adjust the transparency so that these are also visible.

The points are indeed missing because we did not cover any enriched regions from these samples using RNA-seq as background. We specify this in the legend.

- In Figure 1b, why was replicated 2 used?

There was no particular reason, it was just used for illustration.

- Figure 2c : Please label the methods which they are compared to on the right side

We think that this would overload the figure with repetitive text. We explain what is compared in the figure legend.

**2^nd^ round**

**Reviewer 1**

The authors have addressed all my comments. I look forward to the publication of this method, which will be of great value for the community.

**Reviewer 2**

Thanks to the authors who have addressed almost all of the points adequately and overall improved the manuscript. There is only one major point which needs to be addressed, specifically, the description of the method and the underlying assumptions. Please also note, that in the reviewsed document, all variables in the equations are not readable and only viewable as box characters. Changes in the equations could therefore not be reviewed, assuming that equations stayed the same.

1) I thank the author for the discussion. Limiting the comparison to same-sized windows makes sense, clearing up some initial confusion and allows proper discussion of point 3 of my initial comments. The authors added parameters of e.g step sizes in the sliding window approach which improves the quality of the manuscript.

2) I thank the author for looking into the SLBP example. This provides further confirmation for the method. Detecting secondary structures is an interesting case which can be covered by other methods.

3) The description of the method is hard to follow, e.g. “However, as in the foreground sample reads are expected to come largely from bound regions, the unbound regions will be somewhat depleted of reads”. SMI controls are different to ChIP seq background controls, because compared to the IP samples (which are crosslinked, IPed and size selected), SMI controls are crosslinked and size-selected, therefore a sum of RBP-RNA interactions in the selected size. Whereas in ChIP-seq background is to be expected uniform, SMI can contain RBP-RNA interactions of the candidate RBP but in lesser concentrations compared to the rest of the library - as in n/N - m/M. However, the background distribution of the SMI reads can be similar to IP. Are such cases considered?

Thank you for noting that you are not removing RNA types by default.

Thank you for noting that RCRUNCH is only applicable on single replicates. Combining multiple replicates would remove the measure of biological variability. Although out of the scope for this manuscript, it would be nice to incorporate biological replicates in your model framework in future. IDR analysis could be used to combine both replicates as done in van Nostrand 2020 to extract reproducible peaks from e.g. rep1 and rep2 run of RCRUNCH to produce a reproducible set of peaks.

It is not mandatory for this submission to explore the sequencing depth. Just a comment: As eCLIP libraries can have extreme variability in sequencing depth, one could sample down one large library to benchmark this.

4) Thank you for discussing this and adding the Jaccard indices. The reproducibility is worrisome for all methods. It is not clear if this is a property of CLIP, or the protocols. For experimentalists, reproducible sites would be preferential for biologically validation. This would argue for using replicate information for significance testing in CLIP.

5) Thank you for changing the panel. This improves the interpretation quite drastically.

6) Thank you for addressing this.

7) Ok

8) Thank you for clarifying this.

9) Thank you for the discussion.

10) ok

11) see discussion in 3

12) ok

Minor comments:
Ok
